# Principal Components Analysis of Spot Urine Caffeine and Caffeine Metabolites Identifies Promising Exposure Biomarkers of Caffeine and Theobromine Intake in a Cross-Sectional Survey of the United States Population (National Health and Nutrition Examination Survey 2009–2014)

**DOI:** 10.1016/j.cdnut.2025.107441

**Published:** 2025-04-09

**Authors:** Ching-I Pao, Michael E Rybak, Maya R Sternberg

**Affiliations:** National Center for Environmental Health, United States Centers for Disease Control and Prevention, Atlanta, GA, United States

**Keywords:** NHANES, urine, caffeine, metabolites, dietary intake, biomarkers, principal components analysis, PCA

## Abstract

**Background:**

Spot urine concentrations of caffeine and certain caffeine metabolites correlate well with 24-h caffeine intake and have potential use as caffeine exposure biomarkers. However, a high degree of intercorrelation exists among these urine compounds, and their correlations with intake are very similar, making the selection of a single intake biomarker challenging based on bivariate analyses alone.

**Objectives:**

To use principal components (PCs) analysis (PCA) as a dimensionality reduction tool to identify underlying correlation structures in the relationship of caffeine intake with spot urine caffeine and caffeine metabolite concentrations.

**Methods:**

We performed weighted bivariate analyses and a weighted PCA of spot urine concentrations of caffeine and 14 caffeine metabolites, and 24-h intakes of caffeine (foods, beverages, and supplements) and theobromine (foods and beverages) from 7732 participants ≥6 y in the National Health and Nutrition Examination Survey (NHANES) 2009–2014.

**Results:**

Bivariate analyses revealed intercorrelation patterns that effectively divided the urine analytes into 2 groups based on their association with either caffeine or theobromine intake. PCA yielded 2 components (PC1 and PC2) that accounted for 83.0% of the total variance. PC1 (70.9%) showed a positive correlation with all original variables and approximated a weighted sum related to the urine analyte concentrations in the original data. PC2 (12.1%) yielded 2 clusters of correlated variables resembling a weighted contrast of the caffeine and theobromine intake associations observed in the original data. Urine 1-methylxanthine showed the strongest PCA correlation with caffeine intake, followed by 5-acetylamino-6-amino-3-methyluracil, 1-methyluric acid, and 1,7-dimethyluric acid. Urine theobromine and its downstream metabolites showed PCA correlation primarily with theobromine intake, though correlation with caffeine intake was also evident.

**Conclusions:**

With PCA, we were able to identify an underlying correlation structure in the NHANES 2009–2014 data that revealed promising urine exposure biomarkers of caffeine and theobromine intake.

## Introduction

Caffeine is a psychostimulatory purine alkaloid found in plants such as coffee beans, tea leaves, and cocoa beans, entering the diet through foods, beverages, and supplements derived from these sources or as an added ingredient. Most of the global population consumes caffeine daily [1], and in the United States, ∼70% of children and 90% of adults consume caffeine on a given day [2,3]. Because of its neurological effects and dietary prevalence, caffeine has been widely studied as a risk factor in human health [4,5]. Almost all risk factor investigations and population-based estimates of dietary exposure have relied upon dietary intake data to estimate exposure, which can be prone to recall bias, intentional misreporting, inaccuracies in food databases, and variation from natural heterogeneity in food products [6] such as major sources of dietary caffeine (e.g., coffee, tea) [7]. The use of caffeine intake exposure biomarkers holds promise as a complementary and potentially more reliable means of estimating caffeine exposure, but despite this potential, studies identifying and evaluating potential caffeine intake biomarkers have been sparse [[8], [9], [10], [11], [12]].

**FIGURE 1:**
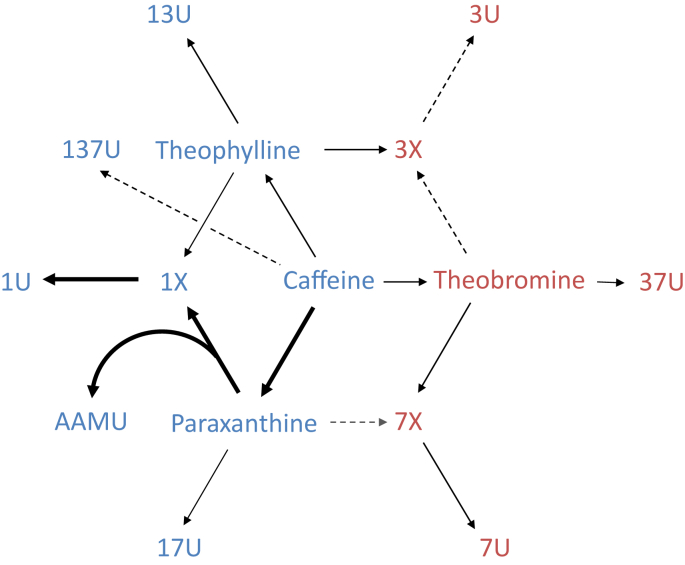
Relationship of caffeine metabolism in humans to correlations observed between spot urine caffeine and caffeine metabolite concentrations and excretion rates with 24-h caffeine intake from foods and dietary supplements in United States persons aged ≥6 y, NHANES 2009–2010. The weight (thickness) of the arrows indicates a relative preference for the indicated metabolism step. Compounds moderately correlated with caffeine intake in the NHANES 2009–2010 [Spearman (ρ) 0.55–0.68, *P* < 0.0001] are shown in blue. Compounds weakly correlated with caffeine intake (ρ = 0.15–0.33, *P* < 0.0001) are shown in red. Intercorrelation among urine analyte concentrations and excretion rates was higher within the groups of moderate and weak intake correlations (ρ = 0.72–0.97, *P* < 0.0001) than between groups (ρ = 0.22–0.58, *P* < 0.0001). AAMU, 5-acetylamino-6-amino-3-methyluracil; NHANES, National Health and Nutrition Examination Survey; 137U, 1,3,7-trimethyluric acid; 13U, 1,3-dimethyluric acid; 17U, 1,7-dimethyluric acid; 1U, 1-methyluric acid; 1X, 1-methylxanthine; 37U, 3,7-dimethyluric acid; 3U, 3-methyluric acid; 3X, 3-methylxanthine; 7U, 7-methyluric acid; 7X, 7-methylxanthine. Reproduced figure and correlation data from reference [8] with permission.

From 2009 through 2014, spot urine caffeine and caffeine metabolite concentration data were included in the Centers for Disease Control and Prevention’s NHANES, a nationally representative cross-sectional survey on the health and nutritional status of the civilian noninstitutionalized United States population. We first reported for the NHANES 2009–2010 survey cycle [8] that spot urine caffeine and caffeine metabolites could be classified into 2 apparent groups based on how strongly their concentrations and excretion rates correlated with 24-h caffeine intake from foods, beverages, and supplements. Spot urine concentrations and excretion rates for caffeine and its metabolites generated via paraxanthine or theophylline showed moderate correlation with intake [Spearman (ρ) = 0.55–0.68, *P* < 0.0001], whereas the remaining urine compounds generated primarily via theobromine showed noticeably lower correlation with intake (ρ = 0.15–0.33, *P* < 0.0001) (Figure 1). In the same study, we also found a higher degree of intercorrelation among analyte concentrations and excretion rates within these 2 apparent groups (ρ = 0.72–0.97, *P* < 0.0001) than across the 2 groups (ρ = 0.22–0.58, *P* < 0.0001) [8]. Since then, a range of studies have used the NHANES urine caffeine and caffeine metabolite excretion data as a collective proximal indicator of caffeine intake to study the association of these measures with health factors that have included hypertension [13], stroke [14], metabolic syndrome [15], bone mineral density [16], asthma and lung function [17], and cognitive performance [18].

Although the correlations we observed for spot urine analytes with caffeine intake in the NHANES 2009–2010 data revealed a group of candidate exposure biomarkers, simplifying the urine analyte-intake relationship by identifying a single urine analyte as an ideal exposure biomarker is worth considering. Accomplishing this based solely on bivariate analyses is difficult given the similarity of the analyte × intake correlations within the group combined with the high intercorrelation between the group analytes. In this study, we revisited the relationship of caffeine intake with spot urine caffeine and caffeine metabolite excretion in the NHANES, using principal components analysis (PCA) as a visualization tool to help with the identification of a single or reduced subset of preferred caffeine exposure biomarkers. PCA is a multivariate procedure [19] that reduces the dimensionality of a dataset by transforming the original variables into a few uncorrelated, orthogonal linear combinations called principal components (PCs) that explain a large amount of the total variability in the dataset. These PCs can then be related back to the original variables to uncover underlying correlation and covariance structures in the original data that may not have been evident in bivariate analyses. We selected PCA because of its potential to reduce the dimensionality of the highly intercorrelated NHANES intake and urine excretion data through variable transformation while providing the ability to visualize the relationship of the uncorrelated, transformed data back to the original variables through various plots. We also expanded upon our earlier work with the NHANES 2009–2010 data [8] in 2 ways: we analyzed all available spot urine caffeine and caffeine metabolite data in the NHANES (2009–2014), and we included dietary theobromine intake as an additional variable for consideration in exposure biomarker identification, given its conspicuous presence in foods and beverages derived from cocoa and tea [20].

## Methods

### Study design and subjects

The NHANES is a cross-sectional survey on the health and nutritional status of the civilian noninstitutionalized United States population conducted by the Centers for Disease Control and Prevention’s National Center for Health Statistics [21]. Since 1999, the NHANES has been conducted in a continuous format divided into 2-y survey cycles. We used data from the NHANES survey cycles in which spot urine caffeine and caffeine metabolite concentration data was available at the time of our analyses, i.e., NHANES 2009–2010, 2011–2012, and 2013–2014. The NHANES makes use of a stratified, multistage probability sample that is representative of the United States population based on age, sex, and race-ethnicity. Survey participants first undergo a home interview during which sociodemographic, dietary supplement use and health-related data are collected. Participants then visit a mobile examination center (MEC) where they undergo a physical examination, blood and urine sample collection, and other assessments, including a 24-h dietary recall of foods and beverages consumed during the 24-h period prior to the interview. All respondents gave their informed consent, and the NHANES protocol was reviewed and approved by the National Center for Health Statistics research ethics review board. Unweighted response rates (relative to the screened sample) for the NHANES 2009–2010, 2011–2012, and 2013–2014 survey cycles ranged from 71.0–79.4% for the interviewed sample and 68.5–77.3% for the examined sample [22].

### Urine samples and laboratory analyses

Spot urine samples were collected from all NHANES 2009–2014 participants ≥6 y who were able to provide a specimen at the time of their MEC visit. Participants were instructed to provide a complete void urine sample as soon as possible upon arrival at the MEC. The date, time, and volume of the urine collected at the MEC were recorded, as well as the date and time of the last urine void prior to the MEC collection. Collected samples were then processed at the MEC to create aliquots for the various urine-based tests performed as part of the NHANES. Urine caffeine and caffeine metabolites were measured on a randomly selected one-third subset of participants in NHANES 2009–2010 (*n* = 2831), 2011–2012 (*n* = 2441), and 2013–2014 (*n* = 2713). For these samples, 1 mL aliquots of urine were transferred to 2 mL polypropylene cryogenic storage vials and frozen immediately. Aliquots were then shipped to our laboratory (Division of Laboratory Sciences, National Center for Environmental Health, Centers for Disease Control and Prevention) on a weekly basis and stored at −70°C or lower until analyzed.

We analyzed spot urine samples by use of liquid chromatography-tandem mass spectrometry [23] for the following compounds: caffeine; paraxanthine; theobromine; theophylline; 1,3,7-trimethyluric acid (137U); 1,3-dimethyluric acid (13U); 1,7-dimethyluric acid (17U); 1-methyluric acid (1U); 1-methylxanthine (1X); 3,7-dimethyluric acid (37U); 3-methyluric acid (3U); 3-methylxanthine (3X); 7-methyluric acid (7U); 7-methylxanthine (7X); and 5-acetylamino-6-amino-3-methyluracil (AAMU). Except for minor changes in procedures and instrumentation over the 2009–2014 NHANES survey cycles [[24], [25], [26]], the general analysis principle for these measurements remained unchanged. For each analysis, 50 *μ*L of urine was first diluted 1:10 with 450 *μ*L of water. 100 *μ*L of the diluted sample was then combined with 120 *μ*L of a 0.2 M sodium hydroxide solution that contained stable isotope labeled internal standards of all analytes. This mixture was incubated for ≥30 min at ambient laboratory temperature to allow the complete conversion of 5-acetylamino-6-formylamino-3-methyluracil, an unstable intermediate compound, to AAMU. Samples were then acidified with 30 *μ*L of 2.0 M hydrochloric acid followed by dilution with 250 *μ*L of a 1:9 methanol:water solution containing 0.1% formic acid to match the sample matrix with the starting mobile phase composition of the analysis step. Samples were then filtered and analyzed by liquid chromatography-electrospray-tandem mass spectrometry in both positive and negative ionization modes. Quantitation was based upon peak area ratios interpolated against an 11-point calibration curve derived from calibrators in synthetic urine. Limits of detection ranged from 3 to 100 nmol/L depending upon the analyte and NHANES cycle [[24], [25], [26]]. We reported results for all analytes in 7984 participants. All results satisfied the requirements of a multi-rule quality control system [27].

### Study variables

Our PCA variables consisted of spot urine concentrations (micromoles/liter) for the 15 analytes identified above [[28], [29], [30]] and 24-h dietary intakes (milligram/day) of caffeine and theobromine from the 24-h dietary recall interview (day 1), which captured the 24-h dietary intake period (midnight to midnight) immediately preceding the MEC visit during which the urine sample was collected. We calculated caffeine intake as the sum of intake from foods and beverages [[31], [32], [33]], nonprescription and prescription dietary supplements, and nonprescription antacids that contain calcium and/or magnesium [[34], [35], [36]]. Theobromine intake consisted solely of intake from foods and beverages [[31], [32], [33]] as theobromine intake data from dietary supplements and antacids were not available in the NHANES 2009–2014. Food and beverage and dietary supplement and antacid intake data were not available for other caffeine-related methylxanthines (e.g., theophylline) in the NHANES 2009–2014.

### Analytic sample

To generate our analytic sample, we considered all MEC-examined NHANES 2009–2014 participants ≥6 y for which urine caffeine and caffeine metabolite measurements, as well as caffeine and theobromine intake data, were available. Unlike our previous study of NHANES 2009–2010 [8], in which we excluded individuals who reported in the past 30 d the use of any prescription medications that might inhibit or induce any of the enzyme systems involved in caffeine metabolism, we made no such exclusions to our analytic sample in the interest of generating a robust PCA model. Our final analytic sample consisted of 7732 study participants (Supplemental Table 1).

### Statistical methods

We used SAS (version 9; SAS Institute Inc) and SUDAAN (version 9.2; RTI International) software to perform bivariate analyses, and RStudio (RStudio, Inc) and the R package “survey” (version 3.35, Thomas Lumley, 2019) to perform a weighted PCA. MEC examination sample weights were used in all our statistical analyses to account for differential nonresponse or noncoverage and to adjust for oversampling. We used Spearman correlation instead of Pearson correlation so that linearizing transformations did not have to be considered. We computed Spearman correlations as the slope of the regression of the standardized ranks for both variables. To account for the complex survey design in the NHANES sample, we used Taylor series linearization to calculate variance estimates to estimate the SE of the slope from the regression. No variance estimation was performed or reported on the outputs of the PCA since our goal was a descriptive analysis. Regression equations [29] accounting for laboratory method changes from the NHANES 2009–2010 cycle to the NHANES 2011–2012 cycle going forward were used to combine the NHANES 2009–2010, 2011–2012, and 2013–2014 spot urine caffeine and caffeine metabolite data into a single dataset by backward-converting the 2011–2012, and 2013–2014 spot urine data to 2009–2010 equivalents.

## Results

### Descriptive and bivariate analyses

We calculated descriptive statistics (medians, 2.5th and 97.5th percentiles) for spot urine caffeine and caffeine metabolite concentrations and 24-h caffeine and theobromine intakes (Supplemental Table 2), Spearman correlations of spot urine caffeine and caffeine metabolite concentrations with 24-h caffeine and theobromine intakes (Table 1), and Spearman correlations among the spot urine caffeine and caffeine metabolite concentrations (Supplemental Table 3) for the combined NHANES 2009–2014 dataset. Median concentrations in the dataset ranged from 0.52 *μ*mol/L (3U) to 58.0 *μ*mol/L (1U). Median analyte spot urine concentrations in descending order were 1U > AAMU > 7X > 3X > 1X > 17U > theobromine > paraxanthine > 7U > 13U > caffeine > theophylline > 37U > 13U > 3U. Urine caffeine, paraxanthine, theophylline, 13U, 17U, 137U, 1X, 1U, and AAMU showed higher correlation with caffeine intake (ρ = 0.57–0.63, *P* < 0.0001) than urine theobromine, 37U, 3X, 7X, 3U, and 7U (ρ = 0.18–0.28, *P* < 0.0001). Correlation with theobromine intake followed a similar but opposite pattern, with higher correlations observed for urine theobromine, 37U, 3X, 7X, 3U, and 7U (ρ = 0.36–0.41, *P* < 0.0001), and little to no correlation observed for urine caffeine, paraxanthine, theophylline, 13U, 17U, 137U, 1X, 1U, and AAMU (ρ = 0.03–0.07, *P* = 0.002–0.03; theophylline *P* = 0.110). Correlation among analyte concentrations was higher within the 2 groups (ρ = 0.71–0.96, *P* < 0.0001) than across the groups (ρ = 0.24–0.59, *P* < 0.0001). Median 24-h caffeine intake in the combined NHANES 2009–2014 data [84.6 mg/d; 95% confidence interval: 76.5, 93.7 mg/d] was ≥10-fold higher than theobromine intake (6.24 mg/d; 95% confidence interval: 4.40, 7.81 mg/d). The correlation between caffeine and theobromine intake was low (ρ = 0.21, *P* < 0.0001).

**TABLE 1:** Spearman correlation of caffeine and theobromine intakes with urine caffeine and caffeine metabolite concentrations in United States persons ≥6 y, NHANES 2009–2014.

Correlation variable	Caffeine intake^1^	Theobromine intake^2^
Urine concentration, ρ (*P)*		
13U	0.63 (*P* < 0.0001)	0.04 (*P* = 0.026)
Theophylline	0.62 (*P* < 0.0001)	0.03 (*P* = 0.11)
Paraxanthine	0.61 (*P* < 0.0001)	0.05 (*P* = 0.013)
17U	0.61 (*P* < 0.0001)	0.05 (*P* = 0.0097)
AAMU	0.61 (*P* < 0.0001)	0.05 (*P* = 0.017)
Caffeine	0.61 (*P* < 0.0001)	0.04 (*P* = 0.0063)
1X	0.60 (*P* < 0.0001)	0.07 (*P* = 0.0019)
1U	0.59 (*P* < 0.0001)	0.06 (*P* = 0.0024)
137U	0.57 (*P* < 0.0001)	0.04 (*P* = 0.014)
3U	0.28 (*P* < 0.0001)	0.36 (*P* < 0.0001)
3X	0.24 (*P* < 0.0001)	0.39 (*P* < 0.0001)
7U	0.23 (*P* < 0.0001)	0.39 (*P* < 0.0001)
7X	0.21 (*P* < 0.0001)	0.40 (*P* < 0.0001)
37U	0.19 (*P* < 0.0001)	0.38 (*P* < 0.0001)
Theobromine	0.18 (*P* < 0.0001)	0.41 (*P* < 0.0001)
Dietary intake, ρ (*P)*		
Caffeine intake	—	0.21 (*P* < 0.0001)

Abbreviations: AAMU, 5-acetylamino-6-amino-3-methyluracil; NHANES, National Health and Nutrition Examination Survey; 137U, 1,3,7-trimethyluric acid; 13U, 1,3-dimethyluric acid; 17U, 1,7-dimethyluric acid; 1U, 1-methyluric acid; 1X, 1-methylxanthine; 37U, 3,7-dimethyluric acid; 3U, 3-methyluric acid; 3X, 3-methylxanthine; 7U, 7-methyluric acid; 7X, 7-methylxanthine.

1 *n* = 7205.

2 *n* = 7220.

### PCA

**FIGURE 2:**
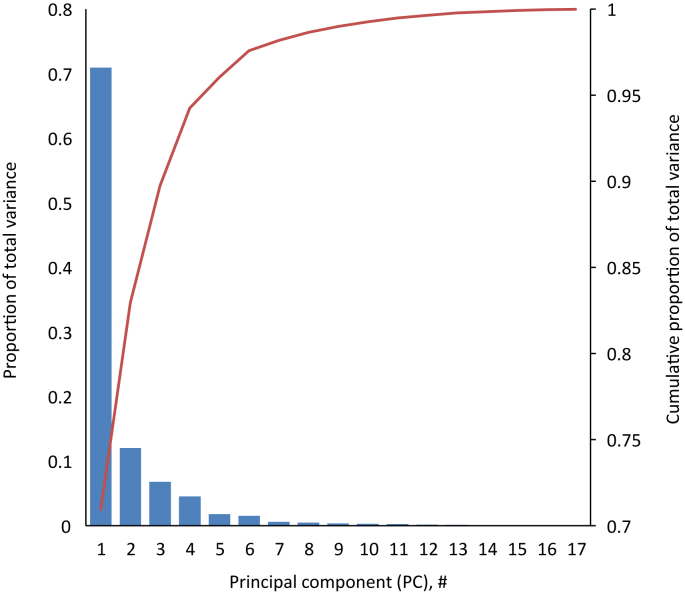
Scree plot of principal component (PC) variance as a function of PC number for PC analysis of spot urine caffeine and caffeine metabolite concentrations in United States persons aged ≥ 6 y, NHANES 2009–2014. The proportion of the total variance described by each PC is indicated by blue bars (left axis), and the cumulative proportion with each PC is indicated by a red line (right axis). The first 2 PCs account for 83.0% of the total variance. NHANES, National Health and Nutrition Examination Survey.

A scree plot of the cumulative variance described by each PC in our PCA is presented in Figure 2 (data presented in Supplemental Table 4). The first (PC1) and second (PC2) components combined accounted for 83.0% of the total variance (PC1: 70.9%; PC2: 12.1%). The scree plot showed an apparent break point at the third component (PC3), with the plot line leveling off beyond this point. PCs whose variance accounts for less than the variance of the original variables (i.e., PC variance < total variance ÷ number of original variables) are typically excluded in PCA interpretation, and although PC3 was slightly above this threshold, we decided in the interest of a simplified 2D data visualization to use only 2 PCs in our data interpretation.

**FIGURE 3:**
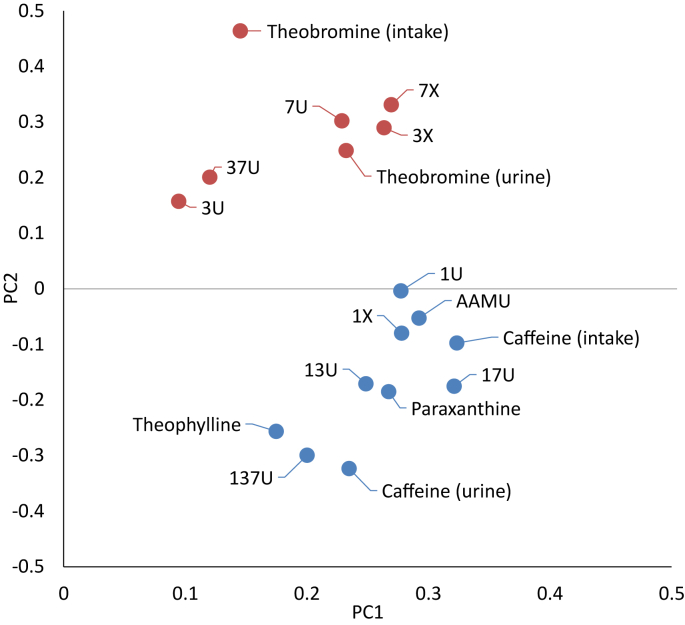
Scatterplot of eigenvector coefficients first (PC1) and second (PC2) principal components for spot urine caffeine and caffeine metabolite concentrations in United States persons aged ≥6 y, NHANES 2009–2014. Compounds showing higher bivariate correlation with caffeine intake [Spearman (ρ) = 0.57–0.63, *P* < 0.0001] compared with theobromine intake (ρ = 0.03–0.07, *P* = 0.002, 0.02; theophylline *P* = 0.110) are indicated in blue. Compounds showing higher bivariate correlation with theobromine intake (ρ = 0.36–0.41, *P* < 0.0001) compared with caffeine intake (ρ = 0.18–0.28, *P* < 0.0001) are indicated in red. AAMU, 5-acetylamino-6-amino-3-methyluracil; NHANES, National Health and Nutrition Examination Survey; PC, principal component; 137U, 1,3,7-trimethyluric acid; 13U, 1,3-dimethyluric acid; 17U, 1,7-dimethyluric acid; 1U, 1-methyluric acid; 1X, 1-methylxanthine; 37U, 3,7-dimethyluric acid; 3U, 3-methyluric acid; 3X, 3-methylxanthine; 7U, 7-methyluric acid; 7X, 7-methylxanthine.

Eigenvector coefficients for PC1 and PC2 are plotted in Figure 3 (data presented in Supplemental Table 5). In a weighted and centered PCA such as ours, the eigenvector coefficients are unit-scaled and represent the correlation between the PCs and original variables. Coefficients for PC1 were positive (0.09–0.32) and ranged from negative to positive for PC2 (−0.32 to 0.46). Caffeine intake showed the highest correlation with PC1, followed by urine 17U, AAMU, 1X, and 1U (0.28–0.32). Theobromine intake showed the greatest positive correlation with PC2, urine caffeine showed the greatest negative correlation, and urine 1U was the least correlated with PC2 (−0.003). Two variable clusters separated by a sign on PC2 were apparent in the plot. Caffeine intake and urine analytes that showed a higher correlation with caffeine intake in our bivariate analyses comprised the negative PC2 cluster, whereas theobromine intake and urine analytes that showed a higher correlation with theobromine intake comprised the positive PC2 cluster.

**FIGURE 4:**
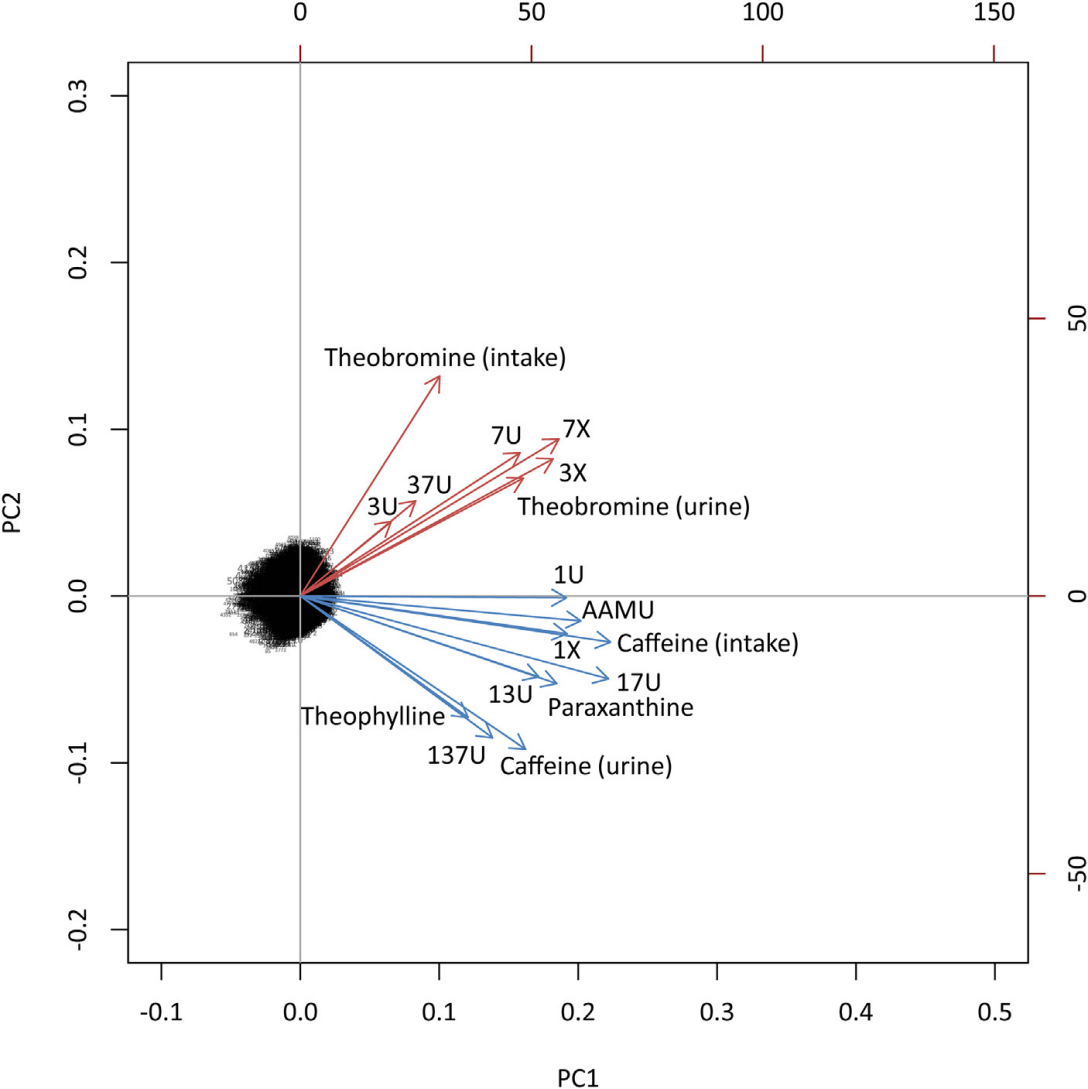
Biplot of principal component (PC) scores and vectors for PC analysis of spot urine caffeine and caffeine metabolite concentrations, and 24-h caffeine intake (foods and dietary supplements) and theobromine intake (foods) in United States persons aged ≥6 y, NHANES 2009–2014. Vectors for caffeine intake and compounds that showed higher bivariate correlation with caffeine intake [Spearman (ρ) = 0.57–0.63, *P* < 0.0001] compared with theobromine intake (ρ = 0.03–0.07, *P* = 0.002, 0.02; theophylline *P* = 0.110 are indicated in blue. Vectors for theobromine intake and compounds showing higher bivariate correlation with theobromine intake (ρ = 0.36,0.41, *P* < 0.0001) compared with caffeine intake (ρ = 0.18–0.28, *P* < 0.0001) are indicated in red. AAMU, 5-acetylamino-6-amino-3-methyluracil; NHANES, National Health and Nutrition Examination Survey; 137U, 1,3,7-trimethyluric acid; 13U, 1,3-dimethyluric acid; 17U, 1,7-dimethyluric acid; 1U, 1-methyluric acid; 1X, 1-methylxanthine; 37U, 3,7-dimethyluric acid; 3U, 3-methyluric acid; 3X, 3-methylxanthine; 7U, 7-methyluric acid; 7X, 7-methylxanthine.

We generated a biplot (Figure 4) visualizing 2 sets of data from our PCA: PC scores, which are points representing the linear combinations of the original data for PC1 and PC2, and PC vectors which describe the covariance of the original variables by taking the eigenvectors (which represent the orientation of each variable on the PC axes) and scaling each to their respective eigenvalues (which represent the variance; PC vector data presented in Supplemental Table 6). The cloud of PC scores appeared to be centered on the plot origin with no obvious distinguishing features other than slight left-skewness on PC1. Because of the relatively large number of observations in our dataset and the absence of any categorical variables in our analysis, we did not attempt to examine the PC scores further for additional visual features. Two clusters of PC vectors separated by a sign on PC2 were observed in the biplot (Figure 4), similar to the clusters observed in the eigenvector plot (Figure 3), but now with added insight in terms of the variance (vector length), correlation (cosine angle between vectors, Supplemental Table 7), and covariance (inner product of vectors, Supplemental Table 8) of the original variables in relation to the PCs. Apart from urine 3U and 37U, the variance was similar across the original variables. Caffeine intake was most correlated (cosine angle → 0°) with urine 1X, followed by several similarly correlated urine analytes (AAMU, 17U, 1U, paraxanthine, 13U). Urine caffeine, theophylline, and 137U were closely correlated with each other but notably less correlated with caffeine intake than 1X, 1U, 13U, 17U, AAMU, and paraxanthine. Theobromine intake was least correlated (cosine angle → 90°) with urine caffeine, theophylline, and 137U. Urine theobromine and its downstream metabolites (3X, 7X, 3U, 7U, 37U) appeared to be closely correlated with each other and, to a lesser extent, with theobromine intake. Caffeine intake was least correlated with urine 3U, 37U, and 7U. No instances of negative correlation (cosine angle > 90°) were observed. Caffeine intake showed the highest covariance (greatest inner product) with urine 17U, followed by AAMU, paraxanthine, 1X, 1U, caffeine, and 13U. Theobromine intake showed the highest covariance with urine 7X, followed by 3X, 7U, and urine theobromine.

## Discussion

In this study, we used PCA to better understand the multivariate relationship among spot urine caffeine and caffeine metabolite concentrations and 24-h dietary caffeine and theobromine intakes in the United States population ≥6 y of age (NHANES 2009–2014). To the best of our knowledge, our study is the first instance of PCA being used to identify potential caffeine and theobromine intake exposure biomarkers in a cross-sectional, nationally representative survey setting. We found that PCA effectively addressed the intercorrelation in these data, capturing 83% of the total variance in the 17-variable dataset in 2 PCs. Our 2-PC model yielded 2 variable clusters consistent with earlier observations in the NHANES 2009–2010 of 2 groups of urine compounds with distinct correlation characteristics in relation to caffeine intake [8], and our PC vector visualization revealed which urine compounds are most associated with caffeine and theobromine intakes in terms of correlation and covariance.

PC1 and PC2 captured 2 key features of the original dataset: the relative magnitude of the intakes and urine analyte concentrations (PC1) and the relative association of the urine analytes with the 2 intakes (PC2). PC1 was positively correlated with all 17 original variables and resembled a weighted sum of these factors. Urine 1U, which showed the highest correlation with PC1 (i.e., similarity in cosine angle with the PC1 axis), had the highest median concentration in the original data, and correlation with PC1 generally decreased (i.e., cosine angle with the PC1 axis increased) with decreasing median concentration. Urine analytes associated with theobromine intake showed a similar association; however, these compounds had nearly indistinguishable cosine angles from one another, and their weighting with PC1 correlation occasionally deviated from their median concentration order. By extension, PC1 also described the relative importance of the underlying metabolic processes generating the urine analytes in relation to caffeine and theobromine intake. In addition to intake, the amount of each analyte found in the urine is also a function of combined metabolic pathway preferences. Caffeine is metabolized primarily via paraxanthine (∼70%), with more than half of urine metabolites expected to be 1X, 1U, and AAMU [37] and decreasing thereafter. This order of metabolic urine disposition preference is similarly associated with decreasing PC1 value. PC2, which yielded 2 clusters of correlated variables, resembled a weighted contrast of the 2 dominant intake sources driving the urine analyte concentrations observed in the original data. Caffeine is the most abundant purine alkaloid in both nature and the diet, followed by theobromine, where other purine alkaloids such as theophylline, paraxanthine, and monomethylxanthines are only found in trace amounts [38]. It is reasonable to assume that caffeine and theobromine intakes are solely responsible for the urine analytes observed, and the dominating contributions of these 2 intakes are likely the underlying cause of the contrasting bivariate correlation patterns that were apparently captured by PC2. Visually, PC2 appeared to “pry” apart the 2 intake associations weighted in PC1, placing the PC vectors for caffeine and theobromine intakes apart with an increasing degree of orthogonality. The correlation among the PC vectors for the urine variables also appeared to mirror the bivariate correlations we observed among the urine analytes in the original urine data.

Our PCA pointed to a stronger correlation between caffeine intake and later-forming urine metabolites such as 1X, 1U, and AAMU, which was not evident in the bivariate data. Bivariate correlation coefficients for caffeine intake with urine caffeine, paraxanthine, theophylline, 1X, 1U, 13U, 17U, and AAMU fell within a favorably high and relatively narrow range (ρ ∼ 0.6). From a cursory standpoint, any 1 of these compounds could serve as a suitable caffeine intake biomarker if bivariate correlation was the sole consideration for selection. In our PCA, however, 1X, 1U, AAMU, and caffeine intake were more closely correlated with PC1 than the other urine analytes. The apparent association of higher overall analyte concentration (i.e., quantitative metabolic importance) with PC1 correlation was likely a factor in the higher PCA correlation observed for these compounds and ultimately should be considered in the biomarker selection process. Downstream metabolites, such as 1X, 1U, and AAMU, are also more polar and have lower urine flow dependence in their renal clearance [39]; however, it is unclear how this would have improved the PCA correlation given that the spot concentrations for these analytes may be more susceptible to urine dilution and no correction (e.g., creatinine, specific gravity, excretion rate) was performed in our study. Differences in median concentration linked to quantitative importance in metabolism [37] also likely explain the higher PCA correlation with caffeine intake observed for urine 13U compared with theophylline in the PCA despite both urine 13U and theophylline having a near-identical correlation with caffeine intake in our bivariate analyses.

Like caffeine intake, theobromine intake also showed a range of bivariate correlation coefficients with the remaining urine analytes (theophylline, 3X, 7X, 3U, and 7U) that fell within a narrow range albeit notably lower (ρ ∼ 0.4) than the analytes associated with caffeine intake. Urine theophylline, 3X, 7X, 3U, and 7U, also appeared to be highly correlated with each other as a group in our PCA but were less correlated with theobromine intake as compared to the PCA correlation of urine 1X, 1U, and AAMU with caffeine intake. Urine theophylline, 3X, 7X, 3U, and 7U, also showed some, albeit weaker, PCA correlation with caffeine intake, consistent with the bivariate correlations observed for these compounds (ρ ∼ 0.2). This shared correlation with both theobromine and caffeine intakes is expected, as a significant proportion (∼20%) of caffeine is metabolized via theobromine, leading to urine disposition that reflects contributions from both intakes with over 75% expected to be recovered as theobromine, 3X, and 7X [37]. The combined contribution of caffeine and theobromine intake to urine theophylline, 3X, 7X, 3U, and 7U excretion ultimately limits their utility as potential biomarkers of theobromine intake. Furthermore, the highly similar PCA correlations observed for these compounds with theobromine intake suggest that urine analytes exhibiting the greatest PCA covariance (i.e., inner product), such as 7X or 3X, rather than the greatest PCA correlation, may best serve this limited role.

Our PCA study is unique to the field of human caffeine intake exposure biomarker investigations. Other studies, including our previous work [8], have examined the intake × exposure biomarker association strictly from a bivariate standpoint. These have included studies in which caffeine was administered under controlled conditions [9] and studies of uncontrolled dietary intake in both the general population [12] as well as specific sub-populations such as pregnant females [10] and young adults [11]. All of these studies found urine caffeine, paraxanthine, and theophylline to be positively associated with caffeine intake; however, only a few studies looked at all 14 caffeine metabolites as part of their analysis [8,11]. Crews et al. [9], after accounting for CYP1A2 phenotypes based on urine metabolite ratios, identified urine paraxanthine, 17U, and 1X as the best predictors of caffeine intake from an analysis panel that also included urine caffeine and 5-acetylamino-6-formylamino-3-methyluracil. Petrovic et al. [12] found that categorical intake of caffeinated coffee was positively associated with 24-h urine caffeine, paraxanthine, and theophylline. Vanderlee et al. [11] looked at all 14 caffeine metabolites as part of their analysis. We are unaware of any previous investigations of potential theobromine intake biomarkers in humans. We know of only 1 other example of PCA performed on the NHANES urine caffeine and caffeine metabolite data but did not include caffeine or theobromine intakes. Zhou and Quin [15] performed a PCA of urine caffeine and caffeine metabolites in the NHANES 2011–2014, examining their association with metabolic syndrome in which similar analyte intercorrelation was observed and a similar 2-PC system was used in a logistic regression analysis that showed a positive association with metabolic syndrome risk.

We believe the key strength of our study was our selection of PCA to address the highly intercorrelated relationship among caffeine and theobromine intakes, metabolism, and urine disposition. This was evidenced by the fact that by using PCA, we were able to simplify these data into 2 interpretable variables (PCs) that described 83% of the total data variance and helped identify potential caffeine intake biomarkers based on PCA correlation. Our use of the NHANES 2009–2014 dataset to this end was also a strength, as our findings were based upon a nationally representative cross-section of the United States population. We acknowledge, however, that our study approach has limitations, and further work in exposure biomarker identification is needed. Although the dietary intake data in the NHANES 2009–2014 is based on a well-validated procedure [40], caffeine intake underreporting may exist in our analysis due to study design (i.e., absence of caffeine and theobromine intake data from prescription and nonprescription medicines, absence of theobromine intake data from dietary supplements in the NHANES 2009–2014) and omissions during the dietary interview process. Major sources of caffeine, such as coffee and tea, may be prone to natural heterogeneity in their caffeine content [7], and emerging caffeine sources may be difficult to capture. Although both the bivariate and PCA correlations show many favorable intakes × urine analyte correlations, the temporal variability of urine caffeine and caffeine metabolite concentrations is largely unknown study, as does the role of correction for urine flow effects (e.g., excretion rate, creatinine adjustment, and specific gravity) [39] to further guide exposure biomarker selection.

## Author contributions

The authors’ responsibilities were as follows – C-IP, MER, MRS: collaboratively designed the research project; C-IP: directly oversaw all laboratory analyses; MRS: performed all statistical analyses; C-IP: wrote the initial manuscript drafts; MER: wrote the final draft for journal submission incorporating author feedback and has primary responsibility for content; and all authors: read and approved the final manuscript.

## Data availability

Datasets described in the manuscript are available publicly and freely at https://www.cdc.gov/nchs/nhanes/index.html.

## Disclaimer

The findings and conclusions in this study are those of the authors and do not necessarily represent the official views or positions of the CDC/Agency for Toxic Substances and Disease Registry or the Department of Health and Human Services.

## Funding

This study was supported by the Centers for Disease Control and Prevention (CDC). All activities associated with this project were funded through regular appropriations acts of the United States government.

## Conflict of interest

The authors report no conflicts of interest.
