# Supplementary material for: Principal Components Analysis of Spot Urine Caffeine and Caffeine Metabolites Identifies Promising Exposure Biomarkers of Caffeine and Theobromine Intake in a Cross-Sectional Survey of the United States Population (National Health and Nutrition Examination Survey 2009–2014)
Source: Curr Dev Nutr. 2025 Apr 9;9(5):107441. doi: 10.1016/j.cdnut.2025.107441 (PMC12083907; doi:10.1016/j.cdnut.2025.107441)
Supplement: Multimedia component 1 [file mmc1.pdf]

Principal components analysis of spot urine caffeine and caffeine metabolites identifies promising exposure biomarkers of caffeine and theobromine intake in a cross-sectional survey of the US population (NHANES 2009–2014)

Ching-I Pao et al.

Supplemental Table 1. Sample sizes for spot urine caffeine by demographic variables in US persons ≥ 6 y, NHANES 2009–2014

**Variable, *n***	**2009–2010**	**2011–2012**	**2013–2014**	**Total**
All	2714	2397	2621	7732
Age (y)				
6–11	381	390	410	1181
12–19	397	385	425	1207
20–39	616	571	602	1789
40–59	677	563	647	1887
≥60	643	488	537	1668
Sex				
Male	1333	1217	1337	3887
Female	1381	1180	1284	3845
Race-ethnicity				
All Hispanic	882	551	660	2093
Non-Hispanic white	1186	772	992	2950
Non-Hispanic black	500	660	581	1741

Principal components analysis of spot urine caffeine and caffeine metabolites identifies promising exposure biomarkers of caffeine and theobromine intake in a cross-sectional survey of the US population (NHANES 2009–2014)
Ching-I Pao et al.

Supplemental Table 2. 2.5th percentile, median, and 97.5th percentiles of urine caffeine and caffeine metabolite concentrations, and caffeine and theobromine intakes in US persons ≥ 6 y, NHANES 2009–2014

**Descriptive variable**[1]	**2.5th percentile**	**Median**	**97.5th percentile**
Urine[2], µmol/L			
1U	4.47 (4.17 to 4.88)	54.0 (51.9 to 58.0)	474 (453 to 505)
AAMU	0.39 (0.17 to 0.63)	52.0 (48.3 to 56.4)	500 (458 to 538)
7X	1.34 (1.18 to 1.56)	43.7 (42.0 to 46.2)	457 (428 to 489)
3X	0.81 (0.65 to 1.07)	27.7 (26.5 to 29.1)	263 (246 to 285)
1X	1.02 (0.95 to 1.10)	26.6 (25.0 to 28.7)	259 (238 to 284)
17U	0.09 (0.07 to 0.13)	24.0 (22.1 to 25.7)	212 (200 to 232)
Theobromine	0.51 (0.38 to 0.64)	18.3 (17.3 to 19.3)	168 (152 to 180)
Paraxanthine	0.12 (0.08 to 0.18)	15.0 (14.1 to 16.0)	95.7 (89.3 to 102)
7U	0.36 (0.30 to 0.46)	14.7 (13.9 to 15.5)	161 (153 to 173)
13U	0.07 (0.06 to 0.09)	6.21 (5.86 to 6.65)	50.9 (48.6 to 55.8)
Caffeine	<0.01	3.24 (2.9 to 3.61)	33.0 (31.5 to 35.5)
Theophylline	0.02 (0.02 to 0.04)	1.57 (1.47 to 1.67)	10.6 (9.73 to 11.2)
137U	0.01 (0.01 to 0.02)	1.36 (1.26 to 1.44)	15.1 (14.0 to 16.4)
37U	0.04 (0.04 to 0.04)	1.03 (1.00 to 1.08)	11.8 (10.5 to 12.7)
3U	<0.01	0.52 (0.49 to 0.56)	6.27 (5.80 to 6.99)
Intake[3], mg/d			
Caffeine intake	0	84.6 (76.5 to 93.7)	628 (580 to 687)
Theobromine intake	0	6.24 (4.40 to 7.81)	287 (267 to 302)

[1] Abbreviations: 137U, 1,3,7-trimethyluric acid; 13U, 1,3-dimethyluric acid; 17U, 1,7-dimethyluric acid; 1U, 1-methyluric acid; 1X, 1-methylxanthine; 37U, 3,7-dimethyluric acid; 3U, 3-methyluric acid; 3X, 3-methylxanthine; 7U, 7-methyluric acid; 7X, 7-methylxanthine; AAMU, 5-acetylamino-6-amino-3-methyluracil.
[2] n = 7732 for all urine analytes.
[3] First day total nutrient intakes. n = 26543 for both caffeine and theobromine.

Principal components analysis of spot urine caffeine and caffeine metabolites identifies promising exposure biomarkers of caffeine and theobromine intake in a cross-sectional survey of the US population (NHANES 2009–2014)
Ching-I Pao et al.

Supplemental Table 3. Spearman correlation matrix of urine caffeine and caffeine metabolite concentrations in US persons ≥ 6 y, NHANES 2009–2014

**Spearman correlation[1,2], *ρ***	**Caffeine**	**Paraxanthine**	**Theophylline**	**1X**	**137U**	**13U**	**17U**	**1U**	**AAMU**	**Theobromine**	**3X**	**7X**	**37U**	**3U**
Caffeine														
Paraxanthine	0.89													
Theophylline	0.90	0.96												
1X	0.77	0.88	0.85											
137U	0.93	0.9	0.90	0.86										
13U	0.8	0.87	0.91	0.93	0.90									
17U	0.87	0.90	0.93	0.92	0.94	0.97								
1U	0.71	0.82	0.81	0.96	0.83	0.94	0.92							
AAMU	0.72	0.83	0.83	0.87	0.83	0.93	0.92	0.89						
Theobromine	0.37	0.46	0.44	0.43	0.43	0.41	0.43	0.41	0.38					
3X	0.32	0.40	0.42	0.53	0.46	0.54	0.52	0.57	0.52	0.87				
7X	0.24	0.37	0.35	0.52	0.39	0.48	0.45	0.54	0.49	0.88	0.96			
37U	0.29	0.38	0.38	0.50	0.44	0.50	0.47	0.52	0.47	0.9	0.93	0.95		
3U	0.34	0.38	0.42	0.53	0.47	0.57	0.54	0.59	0.53	0.76	0.94	0.88	0.87	
7U	0.26	0.35	0.34	0.49	0.40	0.5	0.48	0.57	0.50	0.82	0.94	0.94	0.91	0.91

[1] *n* = 7732 in all cases. *P* < 0.0001 in all cases.
[2] Abbreviations: 137U, 1,3,7-trimethyluric acid; 13U, 1,3-dimethyluric acid; 17U, 1,7-dimethyluric acid; 1U, 1-methyluric acid; 1X, 1-methylxanthine; 37U, 3,7-dimethyluric acid; 3U, 3-methyluric acid; 3X, 3-methylxanthine; 7U, 7-methyluric acid; 7X, 7-methylxanthine; AAMU, 5-acetylamino-6-amino-3-methyluracil.

Principal components analysis of spot urine caffeine and caffeine metabolites identifies promising exposure biomarkers of caffeine and theobromine intake in a cross-sectional survey of the US population (NHANES 2009–2014)
Ching-I Pao et al.

Supplemental Table 4. PCA eigenvalue statistics for urine caffeine and caffeine metabolite concentrations, and caffeine and theobromine intakes in US persons ≥ 6 y, NHANES 2009–2014

Principal component (PC), #	Standard deviation[1]	Proportion of total variance	Cumulative proportion of total variance
PC1	3.06	0.709	0.709
PC2	1.26	0.121	0.830
PC3	0.946	0.0681	0.898
PC4	0.77	0.0451	0.943
PC5	0.486	0.0180	0.961
PC6	0.45	0.0154	0.976
PC7	0.279	0.00591	0.982
PC8	0.248	0.00468	0.987
PC9	0.213	0.00344	0.990
PC10	0.186	0.00263	0.993
PC11	0.174	0.00229	0.995
PC12	0.142	0.00154	0.996
PC13	0.128	0.00124	0.998
PC14	0.105	0.00085	0.999
PC15	0.0951	0.00069	0.9993
PC16	0.0787	0.00047	0.9997
PC17	0.0603	0.00028	1

[1] Standard deviation units are dimensionless.

Principal components analysis of spot urine caffeine and caffeine metabolites identifies promising exposure biomarkers of caffeine and theobromine intake in a cross-sectional survey of the US population (NHANES 2009–2014)
Ching-I Pao et al.

Supplemental Table 5. Principal components analysis eigenvector coefficients[1] for urine caffeine and caffeine metabolite concentrations, and caffeine and theobromine intakes in US persons ≥ 6 y, NHANES 2009–2014

**Original variable[2]**	**PC1**	**PC2**
Urine concentration		
1U	0.277	-0.004
3U	0.095	0.157
7U	0.229	0.302
13U	0.249	-0.171
17U	0.321	-0.175
37U	0.120	0.201
137U	0.200	-0.299
1X	0.278	-0.080
3X	0.264	0.290
7X	0.270	0.331
Theophylline	0.175	-0.256
Paraxanthine	0.267	-0.185
Theobromine (urine)	0.232	0.249
Caffeine (urine)	0.235	-0.323
AAMU	0.292	-0.053
Dietary intake		
Theobromine intake	0.145	0.464
Caffeine intake	0.323	-0.097

[1] Eigenvector coefficients are dimensionless. Only eigenvector coefficients for the first two principal components are shown
[2] Abbreviations: 137U, 1,3,7-trimethyluric acid; 13U, 1,3-dimethyluric acid; 17U, 1,7-dimethyluric acid; 1U, 1-methyluric acid; 1X, 1-methylxanthine; 37U, 3,7-dimethyluric acid; 3U, 3-methyluric acid; 3X, 3-methylxanthine; 7U, 7-methyluric acid; 7X, 7-methylxanthine; AAMU, 5-acetylamino-6-amino-3-methyluracil.

Supplemental Table 6. Principal components analysis biplot vectors[1] for urine caffeine and caffeine metabolite concentrations, and caffeine and theobromine intakes in US persons ≥ 6 y, NHANES 2009–2014

Original variable[2]	PC1	PC2	Vector magnitude
Urine concentration			
1U	0.847	-0.005	0.847
3U	0.289	0.198	0.350
7U	0.699	0.380	0.796
13U	0.760	-0.215	0.789
17U	0.981	-0.220	1.00
37U	0.367	0.252	0.446
137U	0.612	-0.376	0.719
1X	0.849	-0.100	0.855
3X	0.805	0.364	0.883
7X	0.824	0.416	0.923
Theophylline	0.534	-0.322	0.624
Paraxanthine	0.817	-0.233	0.849
Theobromine	0.710	0.313	0.776
Caffeine	0.717	-0.406	0.824
AAMU	0.893	-0.066	0.896
Dietary intake			
Theobromine intake	0.444	0.583	0.733
Caffeine intake	0.988	-0.122	0.996

[1] Vector coordinates and magnitudes are expressed in dimensionless units.
[2] Abbreviations: 137U, 1,3,7-trimethyluric acid; 13U, 1,3-dimethyluric acid; 17U, 1,7-dimethyluric acid; 1U, 1-methyluric acid; 1X, 1-methylxanthine; 37U, 3,7-dimethyluric acid; 3U, 3-methyluric acid; 3X, 3-methylxanthine; 7U, 7-methyluric acid; 7X, 7-methylxanthine; AAMU, 5-acetylamino-6-amino-3-methyluracil.

Supplemental Table 7. Principal components analysis biplot vector cosine angles[1] for urine caffeine and caffeine metabolite concentrations, and caffeine and theobromine intakes in US persons ≥ 6 y, NHANES 2009–2014

Original variable[2]	1U	3U	7U	13U	17U	37U	137U	1X	3X	7X	Theophylline	Paraxanthine	Theobromine (urine)	Caffeine (urine)	AAMU	Theobromine (intake)	Caffeine (intake)
1U																	
3U	34.7																
7U	28.8	5.8															
13U	15.5	50.1	44.3														
17U	12.3	47.0	41.1	3.2													
37U	34.8	0.1	6.0	50.3	47.1												
137U	31.3	65.9	60.1	15.8	19.0	66.1											
1X	6.4	41.1	35.2	9.1	5.9	41.2	24.9										
3X	24.7	10.0	4.2	40.1	37.0	10.1	55.9	31.1									
7X	27.2	7.5	1.7	42.6	39.5	7.7	58.4	33.5	2.5								
Theophylline	30.8	65.4	59.6	15.3	18.5	65.6	0.5	24.4	55.4	57.9							
Paraxanthine	15.6	50.2	44.4	0.1	3.3	50.4	15.7	9.2	40.2	42.7	15.2						
Theobromine (urine)	24.1	10.6	4.7	39.5	36.4	10.7	55.3	30.5	0.6	3.1	54.9	39.7					
Caffeine (urine)	29.2	63.9	58.0	13.8	16.9	64.0	2.0	22.8	53.9	56.4	1.6	13.6	53.3				
AAMU	3.9	38.6	32.7	11.5	8.4	38.7	27.3	2.5	28.6	31.1	26.9	11.7	28.0	25.3			
Theobromine (intake)	53.1	18.4	24.2	68.5	65.4	18.2	84.3	59.4	28.4	25.9	83.8	68.6	29.0	82.3	57.0		
Caffeine (intake)	6.7	41.4	35.6	8.7	5.6	41.5	24.5	0.3	31.4	33.9	24.0	8.8	30.8	22.5	2.8	59.8	

[1] Angles are expressed in degrees.
[2] Abbreviations: 137U, 1,3,7-trimethyluric acid; 13U, 1,3-dimethyluric acid; 17U, 1,7-dimethyluric acid; 1U, 1-methyluric acid; 1X, 1-methylxanthine; 37U, 3,7-dimethyluric acid; 3U, 3-methyluric acid; 3X, 3-methylxanthine; 7U, 7-methyluric acid; 7X, 7-methylxanthine; AAMU, 5-acetylamino-6-amino-3-methyluracil.

Principal components analysis of spot urine caffeine and caffeine metabolites identifies promising exposure biomarkers of caffeine and theobromine intake in a cross-sectional survey of the US population (NHANES 2009–2014)

Ching-I Pao et al.

Supplemental Table 8. Principal components analysis biplot vector covariances[1] for urine caffeine and caffeine metabolite concentrations, and caffeine and theobromine intakes in US persons ≥ 6 y, NHANES 2009–2014

**Original variable**[2]	**1U**	**3U**	**7U**	**13U**	**17U**	**37U**	**137U**	**1X**	**3X**	**7X**	**Theophylline**	**Paraxanthine**	**Theobromine (urine)**	**Caffeine (urine)**	**AAMU**	**Theobromine (intake)**	**Caffeine (intake)**
1U																	
3U	0.244																
7U	0.591	0.277															
13U	0.645	0.177	0.450														
17U	0.833	0.241	0.603	0.793													
37U	0.310	0.156	0.353	0.225	0.305												
137U	0.520	0.103	0.285	0.546	0.683	0.130											
1X	0.720	0.226	0.556	0.667	0.856	0.287	0.558										
3X	0.680	0.305	0.701	0.533	0.710	0.388	0.356	0.647									
7X	0.696	0.321	0.734	0.536	0.717	0.408	0.347	0.658	0.814								
Theophylline	0.454	0.091	0.251	0.475	0.595	0.115	0.448	0.486	0.313	0.306							
Paraxanthine	0.693	0.190	0.483	0.670	0.853	0.241	0.587	0.717	0.573	0.576	0.511						
Theobromine (urine)	0.600	0.267	0.615	0.472	0.628	0.340	0.317	0.572	0.685	0.715	0.279	0.507					
Caffeine (urine)	0.610	0.127	0.347	0.632	0.793	0.161	0.592	0.650	0.429	0.421	0.514	0.680	0.382				
AAMU	0.757	0.245	0.600	0.693	0.891	0.311	0.572	0.765	0.695	0.708	0.498	0.745	0.614	0.667			
Theobromine (intake)	0.373	0.244	0.532	0.212	0.307	0.310	0.052	0.319	0.570	0.608	0.049	0.227	0.498	0.081	0.358		
Caffeine (intake)	0.838	0.262	0.645	0.777	0.997	0.332	0.651	0.851	0.751	0.763	0.567	0.835	0.663	0.758	0.891	0.367	

[1] Variances are expressed in dimensionless units.

[2] Abbreviations: 137U, 1,3,7-trimethyluric acid; 13U, 1,3-dimethyluric acid; 17U, 1,7-dimethyluric acid; 1U, 1-methyluric acid; 1X, 1-methylxanthine; 37U, 3,7-dimethyluric acid; 3U, 3-methyluric acid; 3X, 3-methylxanthine; 7U, 7-methyluric acid; 7X, 7-methylxanthine; AAMU, 5-acetylamino-6-amino-3-methyluracil.
